# 88 Pediatric Postburn Ear Reconstruction of Significant Cartilage Defects

**DOI:** 10.1093/jbcr/irac012.091

**Published:** 2022-03-23

**Authors:** Martin Buta, Matthew DePamphilis, Branko Bojovic, Daniel N Driscoll

**Affiliations:** Massachusetts General Hospital, Boston, Massachusetts; Boston University School of Medicine, Boston, Massachusetts; Massachusetts General Hospital/Shriners Hospitals for Children-Boston, Boston, Massachusetts; Massachusetts General Hospital, Boston, Massachusetts

## Abstract

**Introduction:**

The ear is a protruding appendage with multiple functional and aesthetic implications. Literature indicates that up to 40-60% of facial burns involve the ear. Ear burns with considerable tissue loss and sensory deficits can negatively impact quality of life, psychosocial functioning, and physical health. Successful ear reconstruction mitigates these undesirable outcomes. The complex architecture of the external ear presents a formidable surgical challenge after burn injury, when scar tissue, impaired blood supply, and trauma to cartilage all influence reconstructive options. A lack of materials that truly replicate the characteristics of uninjured elastic cartilage also presents a longstanding surgical dilemma. In this retrospective study, the authors highlight the utility of reconstructive techniques to address significant cartilage deficits, including conchal transposition flap, composite graft, costal cartilage graft, and porous polyethylene implant.

**Methods:**

A retrospective review was conducted on patients aged 0 to 21 years who underwent cartilage framework reconstruction between January 2004 to January 2021 at a specialized pediatric burn center. Medical records from the hospital’s patient database were screened, and 52 patients (60 ears) who met study criteria were identified. Patient demographics, procedural characteristics, and patient outcomes were analyzed.

**Results:**

For helical rim cartilage defects, 20 patients (23 ears) with an average age of 15 ± 4 years underwent a conchal transposition flap, which was associated with no major complications. In cases involving repair of small to medium cartilage deficits, 9 patients (9 ears) with an average age of 13 ± 5 years underwent a composite graft, which was associated with one case of infection. A total of 20 patients (23 ears) with an average age of 13 ± 6 years underwent porous polyethylene implantation, which was associated with two cases of exposure and one case of infection. Of these porous polyethylene cases, 20 ears involved helical rim reconstruction and 3 involved total ear reconstruction. Costal cartilage grafting was performed in 4 patients (5 ears) with an average age of 13 ± 5 years and was associated with one case of infection. Costal cartilage grafting was utilized to reconstruct 2 helical rims and 3 total ears.

**Conclusions:**

In cases of focal cartilage defects or medium-sized helical rim cartilage loss, highly aesthetic results and minimal complication rates can be achieved with composite graft or conchal transposition flap. When presented with large to total helical rim loss or total ear loss, either costal cartilage graft or porous polyethylene implantation is typically necessary.

